# Regional patterns of excess mortality in Germany during the COVID-19 pandemic: a state-level analysis

**DOI:** 10.1098/rsos.250790

**Published:** 2025-11-12

**Authors:** Christof Kuhbandner, Matthias Reitzner

**Affiliations:** ^1^ Department of Human Sciences, University of Regensburg, Regensburg, Germany; ^2^ Department of Mathematics, Osnabrück University, Osnabrück, Niedersachsen, Germany

**Keywords:** excess mortality, COVID-19, German federal states, vaccination, trust in institutions

## Abstract

This study used a rigorous actuarial approach to estimate excess mortality across German federal states during the first three years of the COVID-19 pandemic. Regional trends were analysed alongside associations with state-level indicators: reported COVID-19 deaths and infections, policy stringency, vaccination rates, demographic and socioeconomic factors. Average excess mortality was moderate in the first year, with substantial regional variation. It increased slightly in the second year, with stable regional patterns. In the third year, excess mortality rose sharply, regional differences diminished, and the most affected states shifted, indicating the emergence of a new excess mortality driver. In the first two years, excess mortality strongly correlated with COVID-19 deaths, although reported COVID-19 deaths substantially exceeded excess deaths. Despite rising excess mortality, COVID-19 deaths declined over time. In the third year, only vaccination rate and trust in institutions showed notable associations, with the latter fully mediated by vaccination rate. Higher vaccination rates correlated with larger increases in excess mortality and with smaller declines in COVID-19 deaths and case fatality rates, even after adjusting for prior mortality levels and time-invariant confounders. This robust finding underscores the need for urgent investigation into potential unintended effects of vaccination or other previously neglected mortality drivers.

## 1. Introduction

To comprehensively assess the impact of the COVID-19 pandemic, a key method is to examine all-cause excess mortality (i.e. the difference between observed and expected deaths) during the pandemic years. This is because excess mortality captures not only deaths directly due to COVID-19 but also indirect effects of the pandemic on other causes of deaths and the impact of pandemic-related measures (e.g. Ioannidis *et al.* [1]).

In a recent study, excess mortality in Germany was estimated using advanced actuarial methods incorporating population tables, mortality tables and longevity trends, revealing a striking pattern (Kuhbandner & Reitzner [2]; for similar results, see De Nicola *et al.* [3,4]; Rockenfeller *et al.* [5], Rößler *et al.* [6]; Scherb & Hayashi [7]): While excess mortality in 2020 rose only marginally above pre-pandemic levels, it increased substantially in 2021 and reached extraordinarily high levels in 2022, a pattern also observed in several other countries (e.g. Scherb & Hayashi [7]; Australian Bureau of Statistics [8]; Kye [9]; Pizzato *et al.* [10]). This surprising trajectory raises the question of which factors might explain the pronounced increase in mortality in Germany long after the pandemic began. One approach to addressing this question is to examine the trajectory of excess mortality in the different federal states of Germany. Should substantial variation exist across states, it would allow for an investigation of associations between the rise in excess mortality and state-specific characteristics.

Such a research strategy has been applied in previous studies. For example, Pizzato *et al.* [10] examined excess mortality across European countries during 2020−2023 and found correlations with several country-specific economic factors and, in particular, negative associations with COVID-19 vaccination rates. Similarly, Thum [11] analysed excess mortality in the German federal states in the second half of 2021 and reported a negative correlation with the COVID-19 vaccination rate in a federal state. These findings have been interpreted in the media as evidence of the effectiveness of the vaccinations.

However, drawing valid conclusions from these studies is problematic. In Pizzato *et al.* [10], only total excess mortality over 2020−2023 was analysed. Yet, excess mortality in 2020 cannot be influenced by vaccination, as no vaccinations were available then. In Thum [11], the correlation between excess mortality and vaccination rate was based on vaccination coverage at a single, arbitrary point in time. Analyses based on aggregated periods or arbitrary time points are not informative. Meaningful conclusions require examining relationships between excess mortality and state-specific characteristics at appropriate temporal and regional resolution.

The aim of the present study was to explore the relationship between excess mortality in Germany across the first three pandemic years and several COVID-19 related, sociodemographic and economic factors. Excess mortality was estimated separately for each of the 16 German federal states and each pandemic year, using state-of-the-art actuarial methods based on population tables, life tables and longevity trends (Kuhbandner & Reitzner [2]). State-level characteristics examined included reported COVID-19 deaths and infections, vaccination rates, policy stringencies, gross domestic products as a measure of wealth, poverty rates, mean age, proportions of people in need of care and trust in institutions.

To achieve finer temporal resolution, excess mortality was calculated separately for the first three years of the pandemic (from April to March). This segmentation avoids two disadvantages of calendar-year analysis. First, because COVID-19 only began affecting mortality in Germany from around April 2020 onwards, calendar-year segmentation underestimates the impact of the pandemic on mortality in 2020 compared with subsequent years. Second, because the largest mortality waves occurred around the turn of the year, calendar-year segmentation artificially splits these waves across years, distorting results.

## 2. Summary

Owing to the wide scope of the analyses presented here, the key findings are summarized as a resource for the reader. Each of these findings will be examined in more detail throughout §§3–5.

- *Excess Mortality in Federal States*: A precise estimate for excess mortality in each German federal state for each pandemic year is provided in §4.1 using the methods described in §3.1 and §3.2.
- *Temporal Shift of Excess Mortality*: Excess mortality in Germany transitioned from a moderate and regionally heterogeneous phenomenon in the first two pandemic years to a sharply rising phenomenon in the third pandemic year, with a concurrent reduction in regional variability.
- *Correlational Pattern*: A correlation matrix between excess mortality and state‑specific quantities is derived in §4.2. We observe two main correlational patterns, correlations of excess mortality with COVID‑19 deaths and COVID‑19 infections, and in the second and third pandemic year with COVID‑19 vaccinations.
- *Decoupling Excess Mortality from COVID‑19 Mortality*: A strong positive correlation between excess mortality and reported COVID‑19 deaths is observed in the first two pandemic years, which completely dissolved in the third pandemic year, see §4.2.1. Reported COVID‑19 deaths fell while excess mortality rose dramatically.
- *Paradoxical Vaccination Correlation with Excess Mortality*: A strong positive correlation emerged in the third pandemic year, federal states with higher vaccination rates exhibited significantly larger increases in excess mortality from the second to the third pandemic year, as is shown in §4.2.2. This association held after adjusting for prior mortality levels and time‑invariant confounders
- *Paradoxical Vaccination Correlation with COVID‑19 Quantities*: Higher vaccination rates were also associated with a smaller decline in reported COVID‑19 deaths and a smaller decline in the SARS‑CoV‑2 case fatality rate from the second to the third pandemic year, see §4.2.3.
- *No Evidence for Long COVID or Protective Stringency Policies*: Higher prior SARS‑CoV‑2 infection rates were associated with lower subsequent excess mortality, ruling out Long COVID as a primary driver in §5.4. Furthermore, the stringency of non‑pharmaceutical interventions showed no significant negative correlation with excess mortality in any pandemic year.
- *Role of Institutional Trust*: A positive association between trust in institutions and rising excess mortality is observed in §5.4, which was fully mediated by vaccination rate, suggesting that trust may have influenced outcomes through its effect on vaccine uptake.
- *Scope of Inference*: The reported correlations indicate a correlative relationship between vaccination rates and excess mortality, but not necessarily a causal relationship. They provide a statistical basis for further investigation by medical and health policy experts.

## 3. Material and methods

### 3.1. Mortality probabilities and population tables

The standard method to compute the expected number of deaths in insurance mathematics consists of taking the population table containing the number $l_{x,t}$ of living x-year old males and the number $l_{y,t}$ of living y-year old females, at the beginning of year t, and multiply in a suitable way (see equation (3.2)) by the male and female mortality probabilities $q_{x,t}$ and $q_{y,t}$, respectively, for year t which are contained in a life table. The most recent population tables and life tables are published annually by the German Federal Statistical Office [12,13].

To estimate the excess mortality in a pandemic situation, one calculates the number of expected deaths if there had been no pandemic. Hence, we used the mortality probabilities $q_{x,2019},\;q_{y,2019}$ of the last prepandemic 2017/2019 life table [14] of the Federal Statistical Office of Germany as the base life table. Because there is a clear visible mortality and longevity trend, we used longevity factors $F_{m}(x),F_{f}(y)$ which are taken from the DAV 2004 R [15] to obtain the mortality probabilities $q_{x,t},\;q_{y,t}$ for the pandemic years t=2020,2021 and 2022. This is consistent with our previous investigation [2] concerning the excess mortality for Germany. We refer to [2] for a discussion concerning the use of different life tables, and to [16] for a discussion of the need and the (slightly conservative) choice of longevity factors and the 2017/2019 life table. These considerations lead to German mortality probabilities


(3.1) $$q_{x,t}=q_{x,2019}e^{-(t-2019)\frac{1}{2}F_{m}(x)}\;\text{ and }\;q_{y,t}=q_{y,2019}e^{-(t-2019)\frac{1}{2}F_{f}(y)},$$

for *t* = 2020, 2021, 2022, and 2023.

Before multiplying the population size with the mortality probabilities, one has to take into account the *birthday problem*. Someone dying at age x could have been of age x−1 or x at the beginning of the year, depending on his birthday and the precise date of death. As explained in detail in [2], this leads to


(3.2) $$\begin{array}{lrlrr} & ED_{x,t} & = & \frac{l_{x-1,t}}{2}\;\frac{q_{x-1,t}+q_{x,t}}{2}+\frac{l_{x,t}}{2}\;\frac{q_{x,t}+q_{x+1,t}}{2}. & \end{array}$$

Here $\frac{1}{2}(q_{x-1,t}+q_{x,t})$ is the mortality probability of someone of age (x−1) alive at the beginning of year t; multiplying by $l_{x-1,t}$ yields the total number of deaths in this group, but only half of them are expected to die after their birthday at age x. In the same way, $l_{x,t}\frac{q_{x,t}+q_{x+1,t}}{2}$ is the expected number of deaths of people of age x at the beginning of the year, but only half of them are expected to die before their birthday. Analogous formulas lead to $ED_{y,t}$, and by summation we obtain the total number of expected deaths,


$$ED_{t}=\sum_{x=0}^{100}ED_{x,t}+\sum_{y=0}^{100}ED_{y,t}.$$

The expected number has to be compared to the observed number of deaths ${\hat{d}}_{t}$ in year t.

In our recent paper [2], this method has been used to compute the expected number of deaths $ED_{t}$ for the years t=2020,2021 and 2022 for Germany. Since the publication of this paper, the population table for 2023 was published by the German Federal Statistical Office, and the number of deaths for 2022 and for 2023 has been updated. This allowed us to compute the expected number of deaths $ED_{t}$ for t=2023 and to state the final results for 2022 and 2023. According to [2]—with the mentioned updates—this yielded the following results:


$$\begin{array}{rrrrrrr} ED_{2020}= & 981\;557, & \;ED_{2021}= & 989\;707, & \;ED_{2022}= & 998\;241, & \;ED_{2023}=1\;004\;882,\\ {\hat{d}}_{2020}= & 985\;572, & {\hat{d}}_{2021}= & 1\;023\;687, & {\hat{d}}_{2022}= & 1\;066\;341, & {\hat{d}}_{2023}=1\;028\;206. \end{array}$$

However, looking at calendar years has two major disadvantages. First, since the COVID-19 pandemic only had an impact on mortality from around April 2020 onwards, segmentation into calendar years underestimates the influence of the pandemic on deaths in 2020 compared to subsequent years, where over the entire year COVID-19 has had effects. Second, the strongest waves of deaths in Germany wer typically observed around the turn of the year. Segmentation in the form of calendar years has the disadvantage that these strong waves of mortality are artificially separated and assigned to different year segments, which can lead to distortions.

Fortunately, as shown in our previous paper [2] (see tables 1 and 2), the expected number of deaths can be distributed on to months using the typical behaviour in the years 2010–2019. This opened a possibility to introduce pandemic years, the first $P_{1}$ from April 2020 to March 2021, the second $P_{2}$ from April 2021 to March 2022 and the third $P_{3}$ from April 2022 to March 2023. According to [2]—again suitably updated—we obtained the excess mortalities in these pandemic years.

**Table 1.** State factors $\beta^{{\mathrm{st}}_{\ell }}$ for the German states. The listed first three digits of $\beta_{P_{i}}^{{st}_{\ell }}$ are independent of the pandemic year.

Baden-Württemberg	0.922
Bavaria	0.961
Berlin	0.986
Brandenburg	1.036
Bremen	1.024
Hamburg	0.968
Hesse	0.970
Mecklenburg-Vorpommern	1.074
Lower Saxony	1.025
North Rhine-Westphalia	1.030
Rhineland-Palatinate	1.008
Saarland	1.081
Saxony	0.995
Saxony-Anhalt	1.112
Schleswig-Holstein	1.021
Thuringia	1.062

**Table 2.** Expected and observed number of deaths in the 16 German states.

state	04/2020–03/2021		04/2021–03/2022		04/2022–03/2023	
	exp.	obs.	exp.	obs.	exp.	obs.
Baden-Württemberg	115 231	116 463	116 699	119 701	118 010	126 409
Bavaria	140 645	145 234	142 187	147 897	143 453	153 736
Berlin	37 078	38 671	37 302	37 541	37 676	39 623
Brandenburg	34 500	36 223	35 120	36 555	35 597	37 768
Bremen	8061	8082	8112	8290	8106	9003
Hamburg	18 337	18 607	18 482	18 881	18 583	20 009
Hesse	69 356	72 041	70 061	71 297	70 626	76 320
Mecklenburg-Vorpommern	22 787	22 519	23 272	24 418	23 556	25 237
Lower Saxony	98 431	97 408	99 760	100 318	100 797	110 448
North Rhine-Westphalia	215 195	216 122	217 136	221 380	218 411	237 528
Rhineland-Palatinate	49 619	49 568	50 157	50 967	50 543	54 253
Saarland	13 835	13 890	13 949	14 414	13 999	15 543
Saxony	57 330	65 674	57 365	60 999	57 528	60 982
Saxony-Anhalt	34 029	35 745	34 291	36 136	34 352	37 271
Schleswig-Holstein	36 647	35 610	37 405	37 025	37 998	41 334
Thuringia	30 576	32 204	30 829	33 281	30 865	33 131
total	981 656	1 004 061	992 127	1 019 100	1 000 102	1 078 595


(3.3) $$\begin{array}{rrrrrr} ED_{P_{1}}= & 981\;656, & \;ED_{P_{2}}= & 992\;127, & \;ED_{P_{3}}= & 1\;000\;102,\\ {\hat{d}}_{P_{1}}= & 1\;004\;061, & {\hat{d}}_{P_{2}}= & 1\;019\;100, & {\hat{d}}_{P_{3}}= & 1\;078\;595. \end{array}$$

### 3.2. Excess mortality in the German federal states: systematic bias

In this paper, we applied the above methods to the 16 German states and compared the occurring respective excess mortalities and deficits. To this end, we started with the German population table [13] and the state population tables containing $l_{x,t}^{{\mathrm{st}}_{\ell }}$ for 16 states ${\mathrm{st}}_{\ell }$, ℓ=1,…16, with x=0,…,100, for the years t=2009,…,2023. These tables were provided by the German Federal Statistical Office, and the Statistical Offices of all German states, except for Rhineland-Palatinate, where the Statistical Office of Rhineland-Palatinate claimed that these numbers for the age groups x≥90 are too imprecise to be offered. Owing to the relation


$$l_{x,t}=\sum_{\ell =1}^{16}l_{x,t}^{{st}_{\ell }},$$

we were able to reconstruct the missing numbers of the population table for Rhineland-Palatinate from the population table for Germany giving $l_{x,t}$ and the population tables for the other 15 federal states containing $l_{x,t}^{{\mathrm{st}}_{\ell }}$.

For the years t=2009,…,2023, the German Federal Statistical Office [12] publishes the observed monthly number of deaths ${\hat{d}}_{x,t}^{{\mathrm{st}}_{\ell }}$ for each of the 16 states ${\mathrm{st}}_{\ell }$, ℓ=1,…,16 for the age groups [0,64],[65,74],[75,84] and the age group 85 and over.

At a first step one could use the mortality probabilities of the life table 2017/2019 [14] of the German Federal Statistical Office for all German states. It turns out that the obtained results do not fit to historical data from the years 2009 to 2019. For nearly all German states, the mortality probabilities deviate systematically from the mortality probabilities for the whole of Germany. Accordingly, when using the German-wide mortality probabilities of the life table 2017/2019, the excess mortality in the different federal states is systematically overestimated or underestimated.

To adapt the German mortality probabilities $q_{x,t},\;q_{y,t}$, we introduced *state factors* $\beta^{{\mathrm{st}}_{\ell }}$, ℓ=1,…,16, to obtain state mortality probabilities:


(3.4) $$\begin{array}{lcr} & q_{x,t}^{{\mathrm{st}}_{\ell }}=\beta^{{\mathrm{st}}_{\ell }}q_{x,t}\text{ and }q_{y,t}=\beta^{{\mathrm{st}}_{\ell }}q_{y,t}. & \end{array}$$

A detailed analysis, investigating a timely dependence of the state factors, shows that the highly nonlinear behaviour of the mortality probabilities and the number of deaths is already incorporated in the construction of the life tables and population tables and that the state factors are in fact independent of the year. The state factors $\beta^{{\mathrm{st}}_{\ell }}$ have been estimated to best fit historical data from 2009 to 2019.

### 3.3. Computation of state factors adjusting mortality probabilities

In the same way as described above, we computed the expected number $ED_{x,t}^{{\mathrm{st}}_{\ell }}$ of x-year old male deaths in state ${\mathrm{st}}_{\ell }$ using


(3.5) $$\begin{array}{rl} ED_{x,t}^{{st}_{\ell }}= & \frac{l_{x-1,t}^{{st}_{\ell }}}{2}\;\frac{q_{x-1,t}^{{st}_{\ell }}+q_{x,t}^{{st}_{\ell }}}{2}+\frac{l_{x,t}^{{st}_{\ell }}}{2}\;\frac{q_{x,t}^{{st}_{\ell }}+q_{x+1,t}^{{st}_{\ell }}}{2}\\ = & \beta^{{st}_{\ell }}\left(\frac{l_{x-1,t}^{{st}_{\ell }}}{2}\;\frac{q_{x-1,t}+q_{x,t}}{2}+\frac{l_{x,t}^{{st}_{\ell }}}{2}\;\frac{q_{x,t}+q_{x+1,t}}{2}\right) \end{array}$$

and equation (3.4), where the mortality probabilities are defined in equation (3.1) and the state factors $\beta^{{\mathrm{st}}_{\ell }}$ are to be defined. (For x=0, we set $q_{-1,t}^{{\mathrm{st}}_{\ell }}=q_{0,t}^{{\mathrm{st}}_{\ell }}$*,* and $l_{-1,t}^{{\mathrm{st}}_{\ell }}=l_{0,t+1}^{{\mathrm{st}}_{\ell }}$ if available, and $l_{-1,t}^{{\mathrm{st}}_{\ell }}=l_{0,t}^{{\mathrm{st}}_{\ell }}$ for t=2023.) Analogous formulas lead to $ED_{y,t}^{{\mathrm{st}}_{\ell }}$. Finally summation yielded the total expected number of deaths


$$ED_{t}^{{\mathrm{st}}_{\ell }}=\sum_{x=0}^{100}ED_{x,t}^{{\mathrm{st}}_{\ell }}+\sum_{y=0}^{100}ED_{y,t}^{{\mathrm{st}}_{\ell }}=\beta^{{\mathrm{st}}_{\ell }}{△}_{t}^{{\mathrm{st}}_{\ell }}$$

in year t. If the year t is a leap year, we added an additional day by multiplying the result by $\frac{366}{365}$.

Observe that the numbers ${△}_{t}^{{\mathrm{st}}_{\ell }}$ are given by the life table 2017/19 of the German Federal Statistical Office and by the population tables $l_{x,t}^{{\mathrm{st}}_{\ell }},\;l_{y,t}^{{\mathrm{st}}_{\ell }}$. We defined the state factors $\beta^{{\mathrm{st}}_{\ell }}$ as the solution of the linear regression


(3.6) $$\text{minimize}\;\sum_{\ell =1}^{16}\sum_{t=2012}^{2019}w_{t,\ell }{\left({\hat{d}}_{t}^{{st}_{\ell }}-\beta^{{st}_{\ell }}\Delta_{t}^{{st}_{\ell }}\right)}^{2},$$

where ${\hat{d}}_{t}^{{\mathrm{st}}_{\ell }}$ are the observed total number of deaths in state ${\mathrm{st}}_{\ell }$ in years t. We chose 2012 as the starting year because at the end of 2011 the German Federal Statistical Office evaluated and changed the method of calculating the mortality probabilities and the population tables. The weights $w_{t,\ell }$ should equal the reciprocal of the variance of the number of deaths ${\hat{d}}_{t}^{{\mathrm{st}}_{\ell }}$ to yield the best linear unbiased estimator. The number of deaths is calculated according to equation (3.5), the sum of binomially distributed independent random variables D∼bin(n,p) with parameters $n=\frac{1}{2}l_{x,t}^{{\mathrm{st}}_{\ell }}$ and $p=\frac{1}{2}(q_{x,t}^{{\mathrm{st}}_{\ell }}+q_{x+1,t}^{{\mathrm{st}}_{\ell }})$. Because the mortality probabilities are close to zero, VD=np(1−p)≈np=ED, which is the variance of the Poisson approximation, which we estimated by its realization ${\hat{d}}_{t}^{{\mathrm{st}}_{\ell }}$, hence we put


$$\begin{array}{c} w_{t,\ell }=\frac{1}{{\hat{d}}_{t}^{{\mathrm{st}}_{\ell }}}\approx \frac{1}{VD_{t}^{{\mathrm{st}}_{\ell }}}. \end{array}$$

Finally, we slightly modified this approach: the method does not take account of the already calculated expected total number of deaths $ED_{t}$ for the whole of Germany in the pandemic years stated in equation (3.3). Clearly, $\sum_{\ell }{△}_{t}^{{\mathrm{st}}_{\ell }}=ED_{t}$, i.e. the sum of the not adjusted expected deaths in all states equals the expected number of deaths in Germany, but the minimizers obtained by equation (3.6) are not additive in the sense that $\sum_{\ell }\beta^{{\mathrm{st}}_{\ell }}{△}_{t}^{{\mathrm{st}}_{\ell }}$ would still also equal $ED_{t}$.

The expected number of deaths relies on the observed number of deaths of the last eight years. Clearly, the sum of the fluctuations of the observed number of deaths in the federal states is larger than the fluctuation of the total number of deaths in Germany. In other words, from a statistical point of view, the largest group gives the most reliable results. Thus we calibrated the sum of the expected number of deaths in each state such that the sum equals the expected number of deaths in Germany for each pandemic year. To achieve this, we fixed i∈{1,2,3}, and optimized equation (3.6) for each pandemic year under the constraint


$$\sum_{\ell =1}^{16}ED_{P_{i}}^{{\mathrm{st}}_{\ell }}=\sum_{\ell =1}^{16}\beta_{P_{i}}^{{\mathrm{st}}_{\ell }}{△}_{P_{i}}^{{\mathrm{st}}_{\ell }}=ED_{P_{i}}.$$

From a mathematical point of view, we introduced a Lagrange multiplier and minimized


$$\sum_{\ell =1}^{16}\sum_{t=2012}^{2019}\frac{1}{{\hat{d}}_{t}^{{\mathrm{st}}_{\ell }}}{\left({\hat{d}}_{t}^{{\mathrm{st}}_{\ell }}-\beta_{P_{i}}^{{\mathrm{st}}_{\ell }}\Delta_{t}^{{\mathrm{st}}_{\ell }}\right)}^{2}-\lambda \left(\sum_{\ell =1}^{16}\beta_{P_{i}}^{{\mathrm{st}}_{\ell }}\Delta_{P_{i}}^{{\mathrm{st}}_{\ell }}-ED_{P_{i}}\right).$$

This results in state factors $\beta_{P_{i}}^{{\mathrm{st}}_{\ell }}$ listed in table 1. The state factors $\beta_{P_{i}}^{{\mathrm{st}}_{\ell }}$ turned out to be practically independent of the pandemic year, the difference between $\beta_{P_{i}}^{{\mathrm{st}}_{\ell }}$ for the three pandemic years is at most 0.00018 in all federal states.

**Figure 1.**
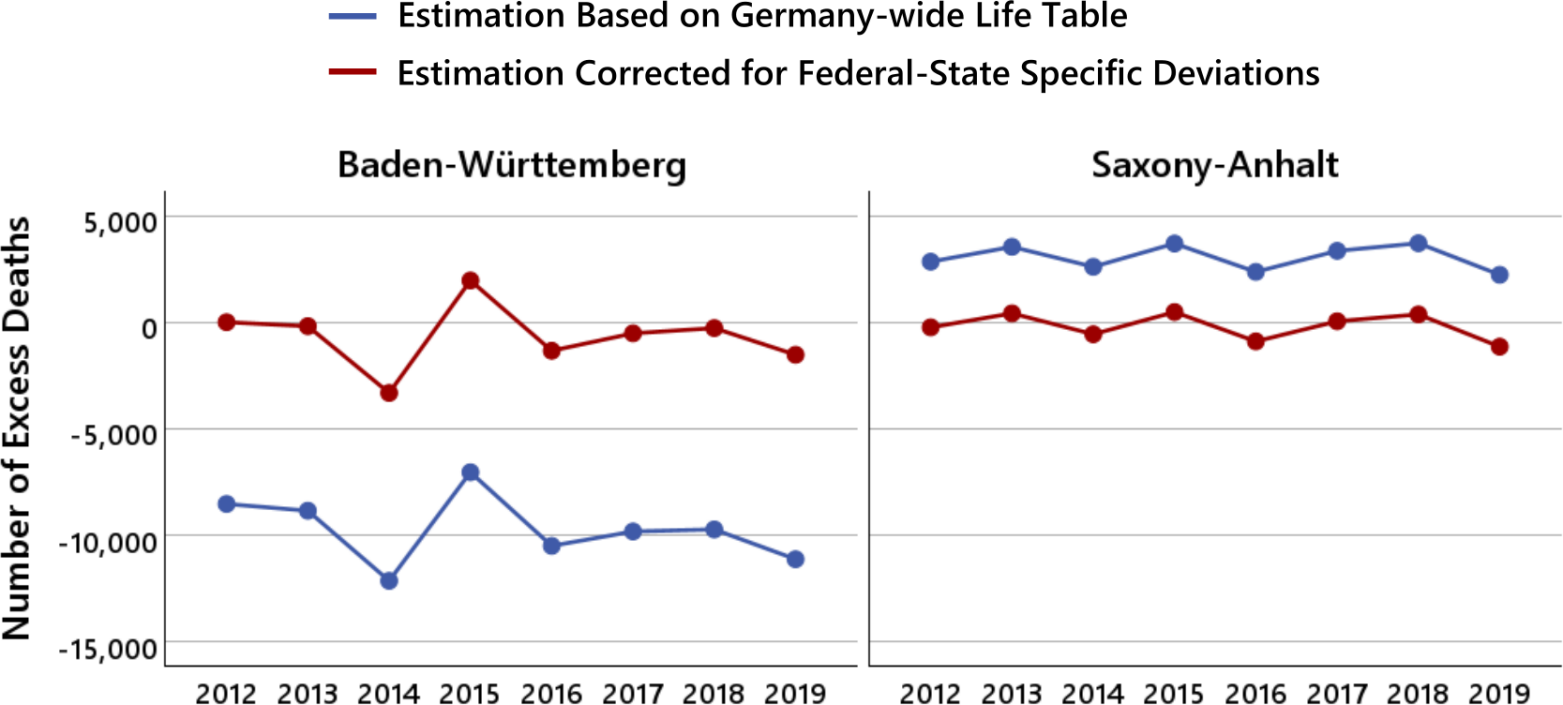
Illustration of the problem of using Germany-wide mortality probabilities of the life table 2017/2019 for the estimates of the state-specific excess mortalities using the data form the federal states of Baden-Württemberg and Saxony-Anhalt that deviate most strongly from the Germany-wide mortality probabilities. The blue dots show the number of estimated excess deaths based on the German-wide mortality probabilities of the life table 2017/2019, the red dots show the number of estimated excess deaths when the estimates are adjusted for the state-specific deviations in mortality probability using the state factors.

We demonstrate the effect of these state factors for the two extreme cases, Baden-Württemberg and Saxony-Anhalt. Figure 1 shows for the prepandemic years 2012−2019, the unadjusted estimated excess deaths ${\hat{d}}_{t}^{{\mathrm{st}}_{\ell }}-{△}_{t}^{{\mathrm{st}}_{\ell }}$ based on the German-wide mortality probabilities of the life table 2017/2019, and the estimated excess deaths ${\hat{d}}_{t}^{{\mathrm{st}}_{\ell }}-ED_{t}^{{\mathrm{st}}_{\ell }}$ when the estimates are adjusted for the federal-state specific deviations in mortality probability using the state factors.

The state factors derived in this way finally allowed us to compute the expected number of deaths $ED_{P_{i}}^{{\mathrm{st}}_{\ell }}$ for each state, and by comparison to the observed number of deaths, the absolute excess mortality $d_{P_{i}}^{{\mathrm{st}}_{\ell }}-ED_{P_{i}}^{{\mathrm{st}}_{\ell }}$ and the relative excess mortality $(d_{P_{i}}^{{\mathrm{st}}_{\ell }}-ED_{P_{i}}^{{\mathrm{st}}_{\ell }})/ED_{P_{i}}^{{\mathrm{st}}_{\ell }}$ in each federal state for the first (April 2020−March 2021), second (April 2021−March 2022) and third (April 2022−March 2023) pandemic years.

To validate the estimated state factors, we calculate the correlations between the state factors and the other state-specific quantities examined in this study (for a description, see below). The state factors are strongly correlated with mean age (r=0.76,p<0.001), proportions of people in need of care (r=0.80,p<0.001) and the gross domestic product (GDP) which represents a measure of the wealth of a federal state (r=−0.67,p=0.005). That is, compared to the German-wide mortality probability reported in the life tables, federal states with a higher average age, a higher proportion of people in need of care, and lower wealth as measured by the GDP show higher mortality probabilities. The three quantities that correlated with the state factors were also highly correlated with each other (mean age/proportion of people in need of care: r=0.85,p<0.001; mean age/GDP: r=−0.88,p<0.001; proportion of people in need of care/GDP: r=−0.76,p<0.001). The state factors were uncorrelated with all COVID-19-related quantities (excess mortality: all p>0.159; COVID-19 deaths: all p>0.481; COVID-19 infections: all p>0.458; COVID-19 measures: all p>0.566; COVID-19 vaccination rates: all p>0.877).

### 3.4. Correlational analysis

To investigate which state-specific quantities covary with the excess mortality observed in a federal state, several key quantities were collected, which are described below. All of the data used in the present study can be downloaded from https://osf.io/xg8eu/.

In a first step, bivariate correlations were calculated between federal state-specific variables and excess mortalities in the three pandemic years; the resulting coefficients are reported in §4.2, and exact two-sided p-values are provided in §5 corresponding to the null hypothesis that the Pearson correlation coefficient equals zero. Where significant associations were observed and potential confounding by time-invariant factors appeared plausible, additional analyses were conducted. To account for stable unobserved differences between states (e.g. demographic structure, health care capacity, baseline health status), change score models were applied. This approach focusses on within-state changes over time, thereby controlling for confounding by unit-level characteristics that remain constant across years. Change scores were calculated as the difference in excess mortality between the first and second pandemic years and between the second and third years. To address residual confounding by prior-year excess mortality (e.g. regression to the mean, mortality displacement), analyses of covariance (ANCOVA) were performed, with the state-specific variable as the primary predictor, change in excess mortality as the dependent variable, and prior-year excess mortality as a covariate. Multicollinearity was assessed using variance inflation factors (VIF) and tolerance statistics, with VIF >5 or tolerance <0.1 considered indicative of potential concern.

#### 3.4.1. Number of COVID-19 deaths

The monthly number of COVID-19 deaths for each German federal state is reported by the Robert Koch Institute (RKI) [17]. The RKI decided to state numbers below four only as ‘<4’ owing to data security reasons . In such cases, we replaced ‘<4’ by two. For a few federal states, no values were reported during a few summer month in 2020. In such cases, the value was set to zero based on the assumption that no COVID-19 deaths occurred in these months. To achieve comparability of the values despite the different population sizes of the different federal states, the number of COVID-19 deaths reported for a federal state was standardized based on the expected number of deaths estimated for that state. That is, we determined for each federal state the percentage of the number of reported COVID-19 deaths relative to the number of expected all-cause deaths. These values reflect the extent to which COVID deaths have occurred in a federal state in relation to the usually expected number of deaths from all other causes of death.

#### 3.4.2. Number of COVID-19 infections

The cumulative number of reported COVID-19 infections in each German federal state was provided daily by the RKI [18]. For the present analyses, we retrieved the cumulative counts at the end of each of the three pandemic years. To allow comparisons across states, the cumulative case counts were standardized by dividing them by the total population of the respective state in the corresponding year. In this manuscript, the term ‘COVID-19 infections’ refers to PCR-confirmed cases as reported by the RKI. This definition does not imply clinically or immunologically confirmed infections and may include uncertainties such as repeated testing of the same individual or selection effects.

#### 3.4.3. Strength of measures taken against COVID-19

In Germany, the containment measures were determined at the level of the individual federal states, so that the intensity of the measures taken varies between individual federal states. Which measures have been imposed over time was recorded on a daily basis by the Federal Ministry for Economic Affairs and Climate on a so-called Corona data platform [19]. The measures imposed in a federal state are summarized in a Corona Severity Index (CSI) that provides information about the dynamics and severity of the imposed measures. The calculation of the CSI is methodically based on the Oxford Stringency Index, that is, the imposed measures are evaluated according to several categories and summed based on a point system that ranges from zero (no measures at all) to 100 (maximal strength). To determine the strength of imposed measures in a federal state in a pandemic year, the mean across the monthly CSI was calculated.

#### 3.4.4. Vaccination rates

The percentage of people vaccinated (first, second and third vaccinations) in each of the German federal states was reported daily by the RKI [20]. To examine the relationship with excess mortality, the vaccination rates reported at the end of each month during the three pandemic years were retrieved. The correlations of the vaccination rates achieved at the end of a month show that the order of the federal states in terms of vaccination rates was very stable over time. In the third pandemic year, the order of the federal states was extremely stable (rate of second vaccinations: all r>0.99; rate of third vaccinations, all r≥0.96), and the order of the federal states for the rates of second and third vaccinations was also very similar (April 2022: r=0.82; since June 2022: all r>0.88). In the second pandemic year, the order of the federal states was also extremely stable (rate of second vaccinations: since August 2021: all r>0.99; rate of third vaccinations: since January 2022: all r>0.98; rates for second and third vaccinations: April 2022: r=0.82; since June 2022: all r>0.88). Owing to this highly similar pattern in vaccination rates across time and types of vaccination, we decided to use the percentage of triple vaccinated people at the end of a pandemic year as an indicator for the vaccination rate in a federal state, and to report the correlations between excess mortality and the monthly rates of double and triple vaccinated people in the electronic supplementary material.

#### 3.4.5. Gross domestic product

The GDP represents a measure of the wealth of a federal state which is in Germany provided in a joint statistics portal of the statistical offices of the federal states [21]. To control for differences in population sizes, the GDP per capita was used which shows a federal state’s GDP divided by its total population. The variations in the GDPs per capita across the federal states in the years 2020, 2021 and 2022 were extremely similar (all r>0.99). Owing to this extremely similar pattern across time, we decided to use the mean GDP per capita across the years 2020–2022.

#### 3.4.6. Poverty rate

To measure poverty in Germany, the German Federal Statistical Office determines the rate of people that are below the poverty risk threshold (i.e. the at-risk-of-poverty rate) for each of the federal states [22]. According to this measurement, people who have less than 60% of the median income of the population are considered to be at risk of poverty. The variations in the at-risk-of-poverty rates across the federal states in the years 2020, 2021 and 2022 were extremely similar (all r>0.96). Owing to this extremely similar pattern across time, we decided to use the mean at-risk-of-poverty rate across the years 2020–2022.

#### 3.4.7. Mean age

The mean age of the population of a federal state for the three pandemic years was calculated based on the age-dependent population size data provided by the German Federal Statistical Office for each year. The variations in mean age across the federal states in the years 2020, 2021, and 2022 were extremely similar (all r>0.99). Owing to this extremely similar pattern across time, we decided to use the mean age across the years 2020 to 2022.

#### 3.4.8. Proportions of people in need of care

The proportions of people in need of care in a federal state is provided by the German Federal Statistical Office [23]. The most current data correspond to the status as of 31 December 2021, and we have used these data.

#### 3.4.9. Trust in institutions

Data on trust in institutions were taken from a large-scale study on the trust in institutions in the German federal states in which approximately 5000 people were surveyed by telephone shortly before the start of the COVID-19 pandemic [24]. In this study, the participants were asked to rate for each of the following institutions individually how strong their trust was in them, using the scale no trust at all/low trust/partial trust/high trust/very high trust: state government, state parliament, federal state government, federal state parliament, political parties, courts, police and public media. The average across all institutions of the respective percentages of people who stated that they had a high or very high trust was used as a measurement of trust in institutions.

## 4. Results

### 4.1. Excess mortality in german states

Using the state factors derived in §3.3, we computed the expected number of deaths for each of the German states ${\mathrm{st}}_{\ell }$, ℓ=1,…,16*,* for the three pandemic years $P_{i}$, i=1,2,3. We compare them in table 2 to the observed values ${\hat{d}}_{P_{i}}^{{\mathrm{st}}_{\ell }}$. The sum over all federal states clearly equals the numbers for Germany already stated in equation (3.3).

The (absolute) excess mortality is the difference between the observed values and the expected values:


$${\hat{d}}_{P_{i}}^{{\mathrm{st}}_{\ell }}-ED_{P_{i}}^{{\mathrm{st}}_{\ell }},$$

and the relative excess mortality is


$$\frac{{\hat{d}}_{P_{i}}^{{\mathrm{st}}_{\ell }}-ED_{P_{i}}^{{\mathrm{st}}_{\ell }}}{ED_{P_{i}}^{{\mathrm{st}}_{\ell }}},$$

which is listed in table 3. For a better comparison to normal years and the usual flue waves, we state in the first row, the empirical s.d. of the last pre-pandemic years and mark those excess mortalities in red which are beyond twice the usual standard deviation.

**Table 3.** Relative excess mortality in the 16 German states.

state	s.d.	04/2020–03/2021	04/2021–03/2022	04/2022–03/2023
Baden-Württemberg	1.43%	1.07%	2.57%	** 7.12%**
Bavaria	1.99%	3.26%	** 4.02%**	** 7.17%**
Berlin	2.09%	** 4.29%**	0.64%	** 5.17%**
Brandenburg	2.22%	** 4.99%**	4.09%	** 6.10%**
Bremen	2.41%	0.26%	2.19%	**11.07%**
Hamburg	1.88%	1.48%	2.16%	** 7.68%**
Hesse	2.18%	3.87%	1.76%	** 8.06%**
Mecklenburg-Vorpommern	2.27%	−1.17%	** 4.92%**	** 7.14%**
Lower Saxony	1.59%	−1.04%	0.56%	** 9.57%**
North Rhine-Westphalia	1.77%	0.43%	1.95%	** 8.75%**
Rhineland-Palatinate	1.68%	−0.10%	1.62%	** 7.34%**
Saarland	2.16%	0.40%	3.34%	**11.03%**
Saxony	1.93%	**14.56%**	** 6.34%**	** 6.00%**
Saxony-Anhalt	1.92%	** 5.04%**	** 5.38%**	** 8.50%**
Schleswig-Holstein	1.81%	−2.83%	−1.02%	** 8.78%**
Thuringia	1.81%	** 5.32%**	** 7.95%**	** 7.34%**

Bolded excess mortality values exceed twice the usual standard deviation.

**Figure 2.**
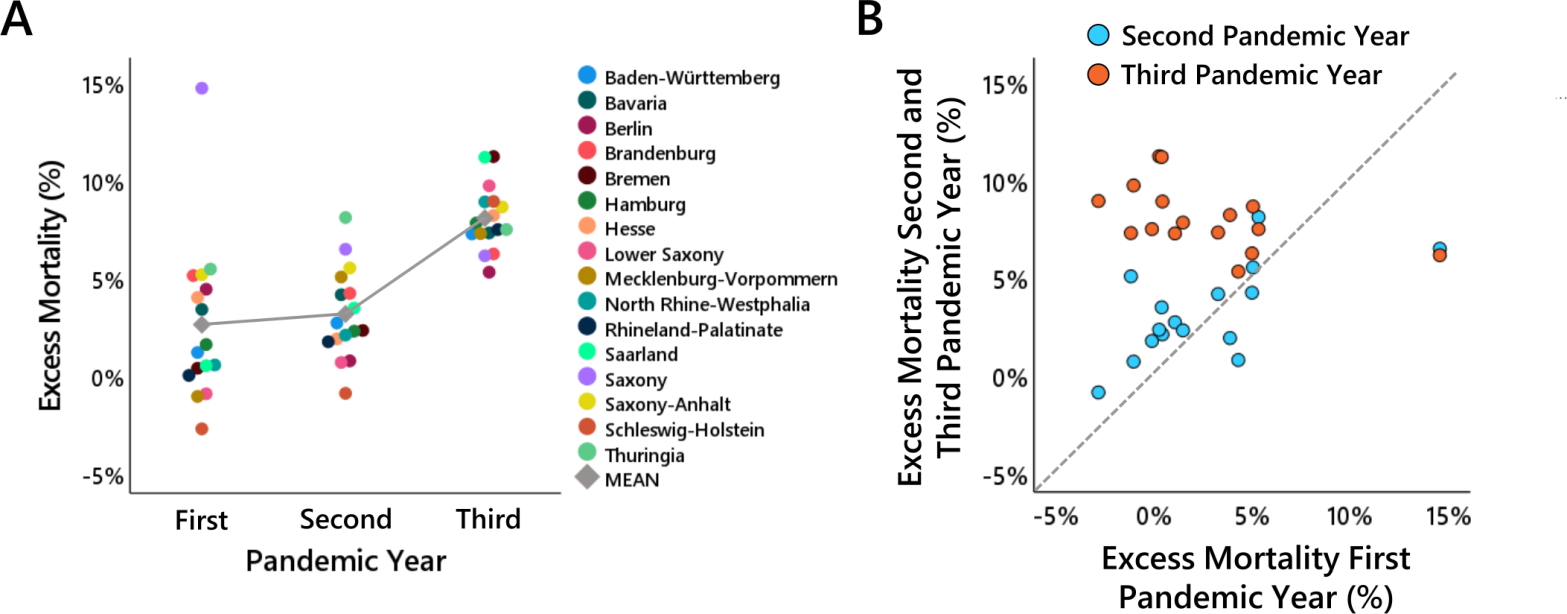
Relative excess mortality in the 16 German federal states. (A) shows for each of the federal states the relative excess mortality in the first, second and third pandemic years. The grey diamond shows the mean across all states and the grey line illustrates the course of mean across the three pandemic years. Data points are jittered slightly along the x-axis to reduce overplotting and improve visibility. (B) shows the temporal stability of the spatial distribution pattern of excess mortality across federal states over the course of the three pandemic years. Excess mortality observed in the first pandemic year is displayed on the x-axes, while excess mortality in the second and third pandemic years is displayed on the y-axes. The dashed line represents the line of equality, indicating the hypothetical case in which excess mortality remained unchanged from the first to the subsequent pandemic years. Greater vertical distance above the dashed line reflects a larger increase in excess mortality in the second and third pandemic years.

Figure 2A depicts the relative excess mortality observed in each federal state for the three pandemic years, represented by coloured dots, along with the overall mean across all states indicated by a grey diamond. Figure 2B illustrates the temporal stability of the spatial distribution pattern of excess mortality across federal states over the course of the three pandemic years.

The observed pattern of excess mortality exhibits several notable features. As can be seen in figure 2A, in the first pandemic year, there was considerable variability across federal states, while the overall level of excess mortality remained relatively moderate (with the notable exception of Saxony). In the second pandemic year, mean excess mortality increased slightly. Yet, as can be seen in figure 2B, the spatial distribution remained largely consistent: federal states that exhibited lower excess mortality in the first pandemic year tended to do so again in the second pandemic year (correlation between excess mortality in the first and second pandemic years: r=0.63, p=0.009), indicating a substantial degree of temporal stability in regional mortality patterns. When examining the increase in excess mortality from the first to the second pandemic year, despite the temporal stability, additional regional differences emerge: the increase in excess mortality tends to be somewhat more pronounced in those federal states that exhibited lower excess mortality in the first year (correlation between excess mortality in the first pandemic year and the increase from the first to the second pandemic year: r=−0.82, p<0.001; after excluding the outlier Saxony in the first pandemic year, r=−0.59, p=0.022).

In the third year of the pandemic, the pattern of excess mortality changed substantially along several dimensions. As can be seen in figure 2A, the average excess mortality increased strongly from the second to the third pandemic year, accompanied by a concurrent reduction in variability across federal states (second pandemic year: s.d.=2.33; third pandemic year: s.d.=1.66). In addition, as can be seen in figure 2B, other federal states are suddenly more or less affected by excess mortality than before, with particularly strong increases in excess mortality being observed in the federal states that exhibited less excess mortality in the first two years of the pandemic (correlation between cumulative excess mortality in the first two pandemic years and excess mortality in the third pandemic year: r=−0.47, p<0.069; correlation between cumulative excess mortality in the first two years and the increase from the second to the third year: r=−0.86, p<0.001).

### 4.2. Correlation matrix

Table 4 lists the correlations between excess mortality and the reported number of COVID-19 deaths and COVID-19 infections, the strength of the measures, the vaccination rates, the GDP, the poverty rate, the mean age, the proportion of people in need of care and the trust in institutions. Two correlation patterns stand out very clearly and are examined in more detail below.

**Table 4.** Correlation matrix.

	1	2	3	4	5	6	7	8	9	10	11	12	13	14	15	16	17	18
1. excess mortality: year 1																		
2. excess mortality: year 2	0.63**																	
3. excess mortality: year 3	−0.52*	−0.25																
4. COVID-19 deaths: year 1	0.96**	0.63**	−0.56*															
5. COVID-19 deaths: year 2	0.78**	0.89**	−0.37	0.84**														
6. COVID-19 deaths: year 3	−0.18	−0.09	0.31	−0.06	0.02													
7. COVID-19 infections: year 1	0.90**	0.58*	−0.42	0.94**	0.77**	−0.03												
8. COVID-19 infections: year 2	0.70**	0.82**	−0.42	0.69**	0.78**	−0.12	0.67**											
9. COVID-19 infections: year 3	−0.72**	−0.63**	0.72**	−0.74**	−0.70**	0.36	−0.72**	−0.64**										
10. strength of measures: year 1	0.17	0.05	0.10	0.27	0.15	0.09	0.24	0.20	0.06									
11. strength of measures: year 2	0.10	0.25	−0.16	0.03	0.07	−0.17	−0.06	0.19	−0.32	−0.16								
12. strength of measures: year 3	−0.09	−0.07	−0.11	−0.06	−0.16	0.15	−0.17	−0.10	0.01	−0.14	0.32							
13. vaccination rate: year 2	−0.80**	−0.78**	0.66**	−0.81**	−0.80**	0.16	−0.70**	−0.80**	0.74**	−0.17	−0.24	0.04						
14. vaccination rate: year 3	−0.82**	−0.81**	0.65**	−0.81**	−0.85**	0.30	−0.68**	−0.79**	0.74**	−0.13	−0.15	0.10	0.96**					
15. gross domestic product	−0.19	−0.39	0.09	−0.09	−0.24	0.56*	0.01	−0.17	0.16	0	0.12	0.09	0.23	0.41				
16. poverty rate	−0.11	−0.04	0.50*	−0.26	−0.19	−0.03	−0.08	−0.32	0.08	−0.40	0.21	−0.17	0.26	0.28	0.11			
17. mean age	0.28	0.65**	−0.01	0.19	0.46	−0.36	0.04	0.37	−0.21	0.06	0.15	0.08	−0.37	−0.52*	−0.88**	−0.10		
18. people in need of care	0.32	0.63**	0.13	0.21	0.44	−0.17	0.16	0.26	−0.18	−0.12	0.07	0.06	−0.36	−0.43	−0.76**	0.26	0.85**	
19. trust in institutions	−0.64**	−0.38	0.59*	−0.61*	−0.43	0.48	−0.49	−0.46	0.60*	−0.42	−0.13	−0.06	0.72**	0.73**	0.37	0.25	−0.36	−0.26

*N* = 16, **p < 0*.*01, *p < 0.05; bold correlation coefficients are significant.”

#### 4.2.1. COVID-19-related correlations with excess mortality

**Figure 3.**
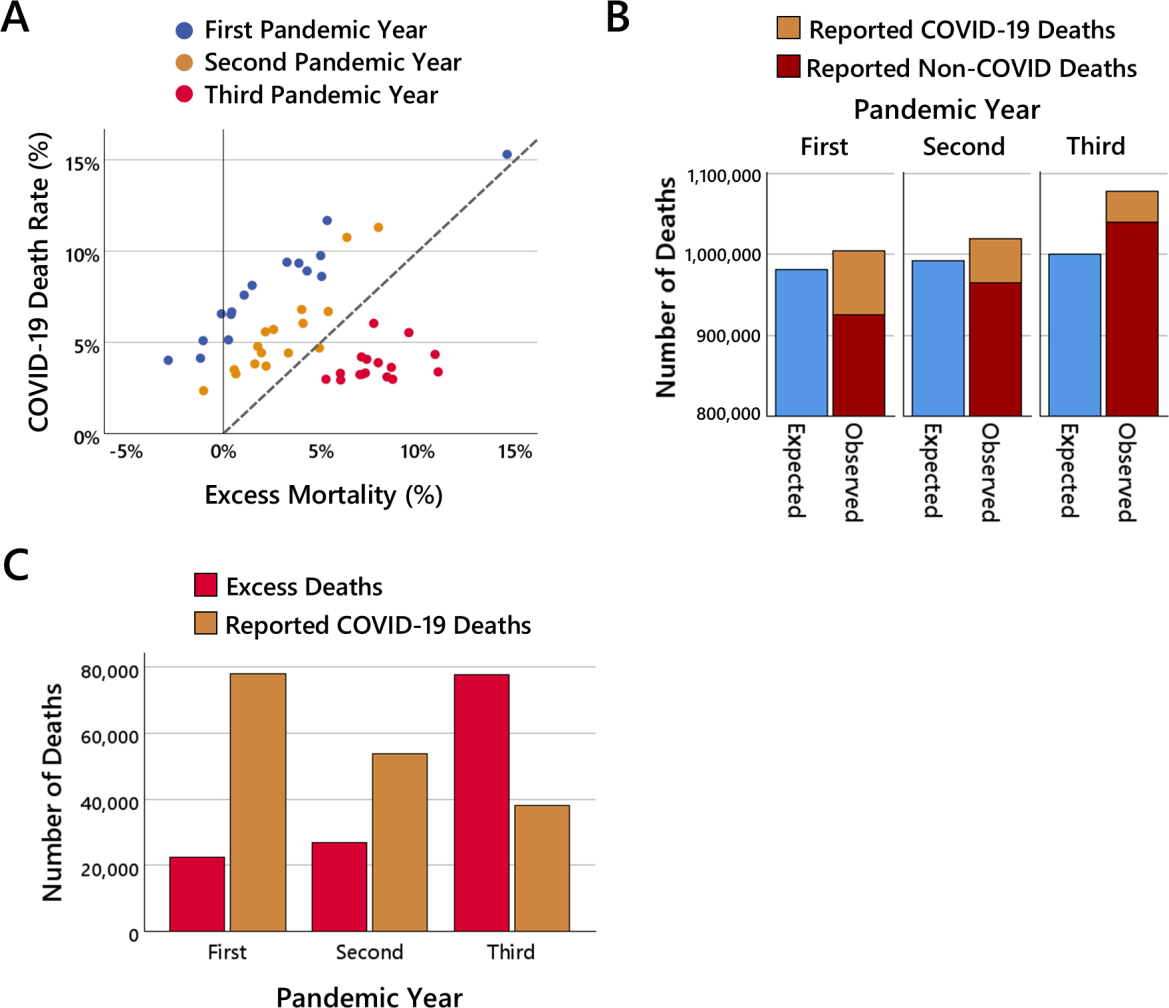
COVID-19-related correlation results. (A) shows the relationship between excess mortality and the number of COVID-19 deaths (in relation to the number of expected deaths, i.e. COVID-19 death rate) for each federal state in the first (blue dots), second (orange dots) and third (red dots) pandemic years. The dashed line indicates what would be the case if the reported number of COVID-19 deaths exactly matched the number of observed excess deaths. (B) shows the total number of expected all-cause deaths (blue bars), the total number of reported COVID-19 deaths (orange bars), and the total number of reported non-COVID deaths (red bars) and (C) shows the number of excess deaths (red bars) and the number of reported COVID-19 deaths (orange bars) across all federal states in the three pandemic years.

The first correlative pattern concerns the association between excess mortality and COVID-19. In the first and second pandemic years, the excess mortality observed in a federal state is highly correlated with the reported number of COVID-19 deaths (first pandemic year: r=0.96, p<0.001; second pandemic year: r=0.89, p<0.001). This pattern changes in the third pandemic year where the correlation between excess mortality and the reported number COVID-19 deaths is no longer significant (r=0.32, p=0.23). To enable further inspection of the correlation pattern, the relevant data are illustrated in more detail in figure 3.

Figure 3A shows the amount of excess mortality and the amount of reported COVID-19 deaths for each federal state in the three pandemic years. What immediately stands out is the strikingly strong association between excess mortality and the reported number of COVID-19 deaths during the first two years of the pandemic. However, upon closer inspection, a peculiar pattern becomes apparent. If the amount of COVID-19 deaths matched the amount of excess mortality, the data points for the federal states should be located along the dashed line. However, the data points are instead consistently well above the dashed line, which means that the number of reported COVID-19 deaths was substantially larger than the number of excess deaths in both the first and the second pandemic years.

This observation is illustrated in figure 3B in more detail where the total numbers of expected deaths, reported COVID-19 deaths, and reported non-COVID deaths across all federal states are shown (the number of non-COVID deaths was determined by computing the difference between the total number of reported all-cause deaths and the officially reported COVID-19 deaths). In the first pandemic year, 981 656 all-cause deaths were expected but 1 004 061 all-cause deaths observed, meaning there were 22 405 more deaths observed than expected. However, at the same time, 78 185 COVID-19 deaths were reported. That is, the number of reported COVID-19 deaths was 3.5 times higher than the number of occurred excess deaths, and the number of reported non-COVID-deaths was only 0.94 times the number of deaths expected without COVID-19. In the second pandemic year, 992 127 all-cause deaths were expected but 1 019 100 all-cause deaths observed, meaning there were 26 973 more deaths observed than expected. However, at the same time, 53 883 COVID-19 deaths were reported. That is, the number of reported COVID-19 deaths was 2.0 times higher than the number of occurred excess deaths, and the number of reported non-COVID-deaths was only 0.97 times the number of deaths expected without COVID-19.

This picture changes fundamentally in the third year of the pandemic where suddenly the number of occurring excess deaths largely exceeds the number of reported COVID-19 deaths. As illustrated in figure 3B, in the third pandemic year, 1 000 102 all-cause deaths were expected but 1 078 595 all-cause deaths observed, meaning there were 78 493 more deaths observed than expected. However, at the same time, only 38 062 COVID-19 deaths were reported. That is, the number of reported COVID-19 deaths was less than half the number of occurred excess deaths, and the number of reported non-COVID-deaths was 1.04 times higher than the number of expected deaths.

Taken together, this reveals a surprising pattern which is illustrated in figure 3C: the number of excess deaths and the number of reported COVID-19 deaths developed in opposite directions across the three pandemic years. From the first to the second pandemic year, the number of excess deaths increased by 4568 while the number of reported COVID deaths decreased by 24 302; and from the second to the third pandemic year, the number of excess deaths increased by 51 520 while the number of reported COVID deaths decreased by 15 821.

Note that excess mortality in the second pandemic year is significantly correlated with COVID-19 deaths in the first year, and excess mortality in the first pandemic year is significantly correlated with COVID-19 deaths in the second year. It seems that the combined effects of increasing excess mortality and decreasing COVID-19 deaths create a complex pattern that asks for further investigations which are beyond this contribution. In the third pandemic year, both quantities are nearly uncorrelated apart from the negative correlation between excess mortality with COVID-19 deaths in the first year.

As for COVID-19 infections the picture is nearly the same. The correlation pattern of excess mortality with COVID-19 infections follows closely the pattern with COVID-19 deaths in the first two pandemic years. In the third pandemic year the pattern changes slightly, the negative correlation between COVID-19 infections in the third pandemic year with excess mortality in the first two pandemic years, and the positive correlation with excess mortality in the third pandemic year, becomes significant.

#### 4.2.2. Vaccination-related correlations with excess mortality

**Figure 4.**
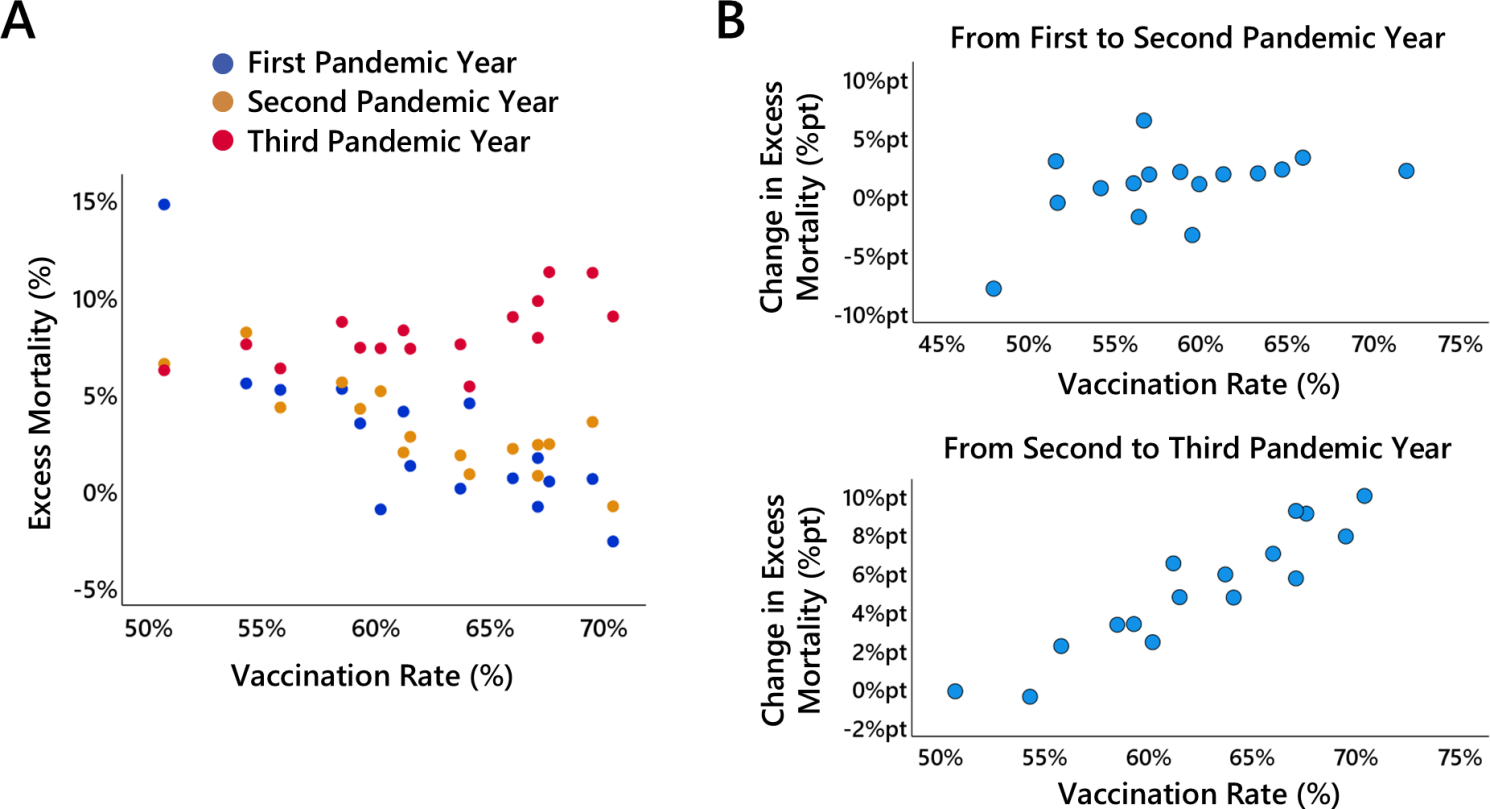
Vaccination rate and excess mortality. (A) shows the relationship between relative excess mortality and the vaccination rate for each federal state in the first (blue dots), second (orange dots) and third (red dots) pandemic years. (B) shows the relationship between the change in excess mortality from the first to the second pandemic year and the vaccination rate for each federal state, (C) shows the relationship between the change in excess mortality from the second to the third pandemic year and the vaccination rate for each federal state.

The second correlation pattern that stands out concerns the associations between vaccination rates and excess mortality as well as the number of reported COVID-19 deaths and infections. Although vaccinations could not yet have exerted any relevant effect during the first year of the pandemic, a negative correlation is observed between excess mortality in the first pandemic year and the vaccination rate reached in the second pandemic year (r=−0.80, p<0.001), indicating that federal states with lower excess mortality in the first pandemic year reached higher vaccination rates in the second year. Precisely the same relationship is found between excess mortality in the second pandemic year and the vaccination rate reached in the second pandemic year (r=−0.78, p<0.001). In the third year of the pandemic, this pattern changes fundamentally. The vaccination rate is now positively correlated with excess mortality (r=0.65, p=0.006). To enable further inspection of the correlation pattern, the relationship between vaccination rate and excess mortality is illustrated in more detail in figure 4.

Figure 4A shows the relationship between the vaccination rate (end of the third pandemic year; note that the vaccination rates at the end of the second and third years were highly correlated, r=0.96) and the amount of excess mortality in the three pandemic years. What is clearly observable is the nearly identical relationship between vaccination rate and excess mortality in the first two pandemic years, as well as the fundamental change in the relationship in the third pandemic year.

The nearly identical association pattern between vaccination rates and excess mortality in the first and second pandemic years strongly suggests the influence of a time-invariant third variable. This confounding factor probably contributed both to elevated excess mortality in certain federal states in the first two pandemic years and to lower vaccination uptake in those same regions. This interpretation is supported by the fact that vaccinations could not have exerted any relevant effect on mortality during the first pandemic year. To control for time-invariant unobserved third variables, a change score model was applied. The associations between vaccination rates and the change in excess mortality from the first to the second, and from the second to the third pandemic year are shown in figure 4B.

The change score analysis reveals a marginally significant positive correlation between vaccination rate and the change in excess mortality from the first to the second pandemic year (r=0.45, p=0.081). However, after excluding Saxony which constituted an outlier in the first pandemic year, this association diminishes (r=0.18, p=0.527). A markedly stronger positive correlation is observed between vaccination rate and the change in excess mortality from the second to the third pandemic year (r=0.93, p<0.001; note that Saxony was not an outlier in the second pandemic year). This indicates that the increase in excess mortality from the second to the third pandemic year was greater in states with higher vaccination rate. Although a change score model accounts for all time-invariant confounders that may simultaneously affect both vaccination rates in the second and third pandemic years and excess mortality across all three years, a residual confounding factor may still be present: excess mortality itself. Specifically, excess mortality observed in the second and third pandemic years may partially depend on the level of excess mortality experienced in prior years. This could reflect regression to the mean, or alternatively, mortality displacement effects, whereby regions with higher early excess mortality experienced a depletion of vulnerable individuals, thereby reducing subsequent excess mortality.

To adjust for the effects of prior-year excess mortality, the ANCOVA model was used. In the present analysis, change scores were used as the dependent variable in a linear regression model, with vaccination rate as the primary predictor and prior-year excess mortality included as a covariate to account for differences in prior-year excess mortality between federal states.

For the change in excess mortality from the first to the second pandemic year, the analysis showed that vaccination rate (second pandemic year) was a significant negative predictor of the increase in excess mortality (β=−0.57; s.e.=0.11; 95% CI [−0.54,−0.06]; t(13)=−2.71; p=0.018), after adjusting for prior-year excess mortality, which was itself a significant covariate (β=−1.27; s.e.=0.16; p<0.001). Accordingly, when controlling for prior-year excess mortality, the previously observed positive association was reversed, indicating that higher vaccination rates were associated with smaller increases in excess mortality.

However, it is important to note that the vaccination rate and excess mortality in the first pandemic year were substantially correlated (r=−0.80), raising concerns about multicollinearity. When predictors share a large proportion of variance, it becomes difficult to determine the extent to which the observed effect can be uniquely attributed to either predictor. Multicollinearity diagnostics indicated no critical concern (VIF=2.74; tolerance =0.36); however, the regression coefficients should nonetheless be interpreted with caution owing to the substantial correlation between the predictors. When Saxony, identified as an outlier in the first pandemic year, was excluded from the analysis, the negative association between vaccination rate and excess mortality increase remained significant (β=−0.75; s.e.=0.13; 95% CI [−0.58,−0.03]; t(12)=−2.40; p=0.034).

For the change in excess mortality from the second to the third pandemic year, the analysis showed that vaccination rate (third pandemic year) remained a highly significant positive predictor of the increase in excess mortality (β=0.68; s.e.=0.08; 95% CI [0.20,0.56]; t(13)=4.57; p<0.001), after adjusting for prior-year excess mortality, which was itself a marginally significant covariate (β=−0.31; s.e.=0.20; p=0.055). As before, the correlation between vaccination rate and prior-year excess mortality was relatively high (r=−0.81), and multicollinearity diagnostics indicated no critical concern (VIF=2.91, tolerance =0.34). Accordingly, prior-year excess mortality was a negative predictor of the subsequent increase in excess mortality, whereas vaccination rate emerged as an additional and notably strong positive predictor.

When including total excess mortality in the first two pandemic years as a covariate to account for differences in excess mortality in both previous pandemic years between federal states, the strong positive association between vaccination rate and excess mortality increase was significant as well (β=0.83; s.e.=0.13; 95% CI [0.19,0.74]; t(13)=3.68; p=0.003). In this case, the correlation between vaccination rate and prior-year excess mortality was very high (r=−0.90), and multicollinearity diagnostics indicated potential concerns (VIF=5.09, tolerance =0.20). Therefore, interpretation of individual regression coefficients should be made with caution owing to potential instability introduced by shared variance.

#### 4.2.3. Vaccination-related correlations with COVID-19

**Figure 5.**
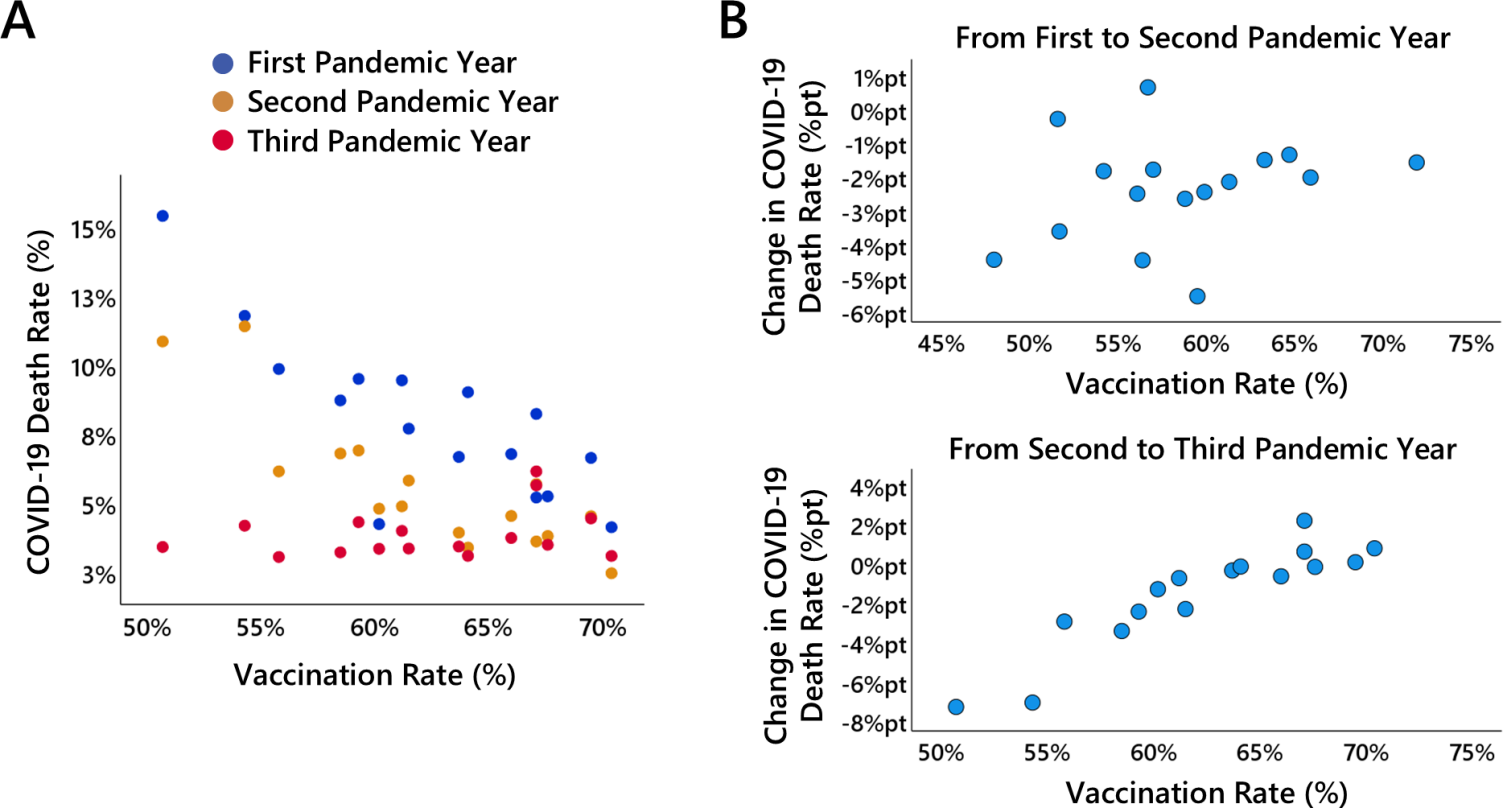
Vaccination rate and COVID-19 deaths. (A) shows the relationship between the number of COVID-19 deaths (in relation to the number of expected deaths, i.e. COVID-19 death rate) and the vaccination rate for each federal state in the first (blue dots), second (orange dots) and third (red dots) pandemic years. (B) shows the relationship between the change in the COVID-19 death rate from the first to the second pandemic year and the vaccination rate for each federal state, (C) shows the relationship between the change in the COVID-19 death rate from the second to the third pandemic year and the vaccination rate for each federal state.

As shown in figure 5, a similar correlational pattern as observed for the association between vaccination rates and excess mortality emerges for the association between vaccination rates and the number of reported COVID-19 deaths and COVID-19 infections.

The vaccination rate in a federal state reached in the second pandemic year was already negatively correlated with the numbers of reported COVID-19 deaths and infections in the first pandemic year (COVID-19 deaths: r=−0.81, p<0.001; COVID-19 infections: r=−0.70, p=0.002), and to the same extent in the second pandemic year (COVID-19 deaths: r=−0.80, p<0.001; COVID-19 infections: r=−0.80, p<0.001), and the pattern changed fundamentally in the third pandemic year where positive correlations were observed (COVID-19 deaths: r=0.30, p=0.254; COVID-19 infections: r=0.74, p=0.001).

A change score analysis reveals a non-significant positive correlation between vaccination rate and the change in the number of reported COVID-19 deaths from the first to the second pandemic year (r=0.23, p=0.386), and a markedly stronger positive correlation between vaccination rate and the change in the number of reported COVID-19 deaths from the second to the third pandemic year (r=0.91, p<0.001). This indicates that the number of reported COVID-19 deaths decreased less from the second to the third pandemic year the higher the vaccination rate in a federal state. In fact, when dividing the federal states at the median vaccination rate, the group with higher vaccination rates showed, on average, an increase (+0.14 %pt) rather than a decrease in reported COVID-19 deaths.

After adjusting for differences in the COVID-19 death rate in the prior year, vaccination rate in the second pandemic year was a non-significant negative predictor of the increase in COVID-19 deaths from the first to the second pandemic year (β=−0.56; s.e.=0.10; 95% CI [−0.36,0.06]; t(13)=−1.50; p=0.157). The prior-year COVID-19 death rate emerged as a significant covariate (β=−0.98; s.e.=0.20; p=0.021). For the change in the COVID-19 death rate from the second to the third pandemic year, after adjusting for differences in the COVID-19 death rate in the first two pandemic years, vaccination rate (third pandemic year) remained a significant positive predictor of the increase in COVID-19 deaths (β=0.48; s.e.=0.09; 95% CI [0.04,0.41]; t(13)=2.57; p=0.023). The COVID-19 death rate in the two previous years emerged as a significant covariate (β=−0.50; s.e.=0.09; p=0.018). Thus, the COVID-19 death rate in the two previous years was a negative predictor of the subsequent increase in the COCID-19 death rate, whereas vaccination rate emerged as an additional positive predictor. Note that vaccination rate and the COVID-19 death rates in the first and the two first pandemic years were substantially correlated (r=−0.81,−0.86), raising concerns about multicollinearity. However, multicollinearity diagnostics indicated no critical concerns (VIF=2.90,3.90; tolerance =0.34,0.26).

**Figure 6.**
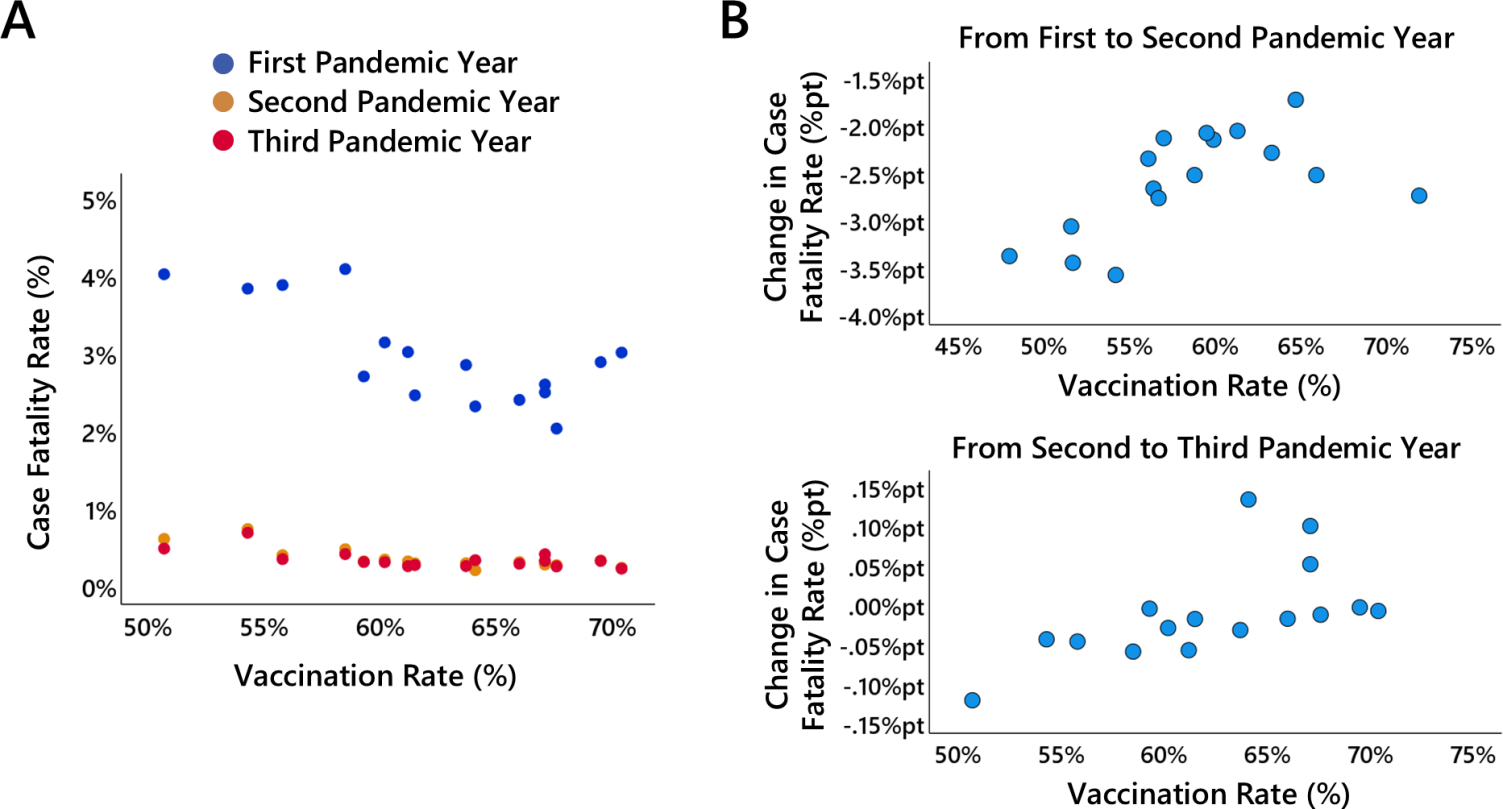
Vaccination rate and case fatality rate. (A) shows the relationship between the case fatality rate and the vaccination rate for each federal state in the first (blue dots), second (orange dots) and third (red dots) pandemic years. (B) shows the relationship between the change in the case fatality rate from the first to the second pandemic year and the vaccination rate for each federal state, (C) shows the relationship between the change in the case fatality rate from the second to the third pandemic year and the vaccination rate for each federal state.

As shown in figure 6, a similar pattern was observed for the case fatality rate, defined as the number of reported COVID-19 deaths relative to the number of reported SARS-CoV-2 infections. Notably, even in the first pandemic year, when vaccinations could not yet have exerted any relevant effect, a negative correlation emerged between subsequent vaccination rates and case fatality rates (r=−0.64; p=0.007). This negative association was comparable in magnitude to the correlations observed in the second and third pandemic years (r=−0.70; p=0.002 and r=−0.62; p=0.010, respectively).

Owing to substantial underreporting of SARS-CoV-2 infections during the first pandemic year, changes in case fatality rates from the first to the second year are difficult to interpret. Consequently, the change score analysis was restricted to the second and third pandemic years. The analysis revealed a significant positive correlation between vaccination rate and the change in the case fatality rate from the second to the third pandemic year (r=0.60, p=0.014). This indicates that the decline in case fatality rate between the second and third pandemic years was less pronounced in federal states with higher vaccination rates. In fact, when dividing the federal states at the median vaccination rate, the group with higher vaccination rates showed, on average, an increase (+0.02 %pt) rather than a decrease in the case fatality rate. Given the lack of a plausible rationale for an influence of prior-year case fatality rate on subsequent-year values, no covariance analysis was performed in this instance.

## 5. Discussion

The objective of this study was to implement a methodologically rigorous approach for estimating excess mortality in the individual German federal states, drawing on the state-of-the-art actuarial method (Kuhbandner and Reitzner [2]). Using this approach, the regional distribution and trajectory of excess mortality over the first three years of the COVID-19 pandemic were analysed, and the association with several key state-level indicators was explored.

The excess mortality results revealed substantial variation across federal states, both in the initial extent of excess mortality and in its trajectory over the three years of the pandemic. Average excess mortality was only moderate in the first year of the pandemic. However, substantial variation was observed across federal states. One state (Saxony) experienced exceptionally high excess mortality, whereas some states (Lower Saxony, Mecklenburg-Western Pomerania and Schleswig-Holstein) even showed a mortality deficit. At first glance, the observation of a mortality deficit in some federal states may appear surprising. However, such findings are not uncommon. A recently published study on excess mortality across 30 high-income countries reported a mortality deficit in 12 countries in 2020 and in 16 countries in 2021 (De Nicola & Kauermann [25]).

In the second year of the pandemic, excess mortality increased only slightly on average. A distribution pattern across federal states similar to that observed in the first pandemic year was found: states that had experienced lower excess mortality in the first pandemic year continued to show comparatively lower levels in the second pandemic year, with a slight regression to the mean—those that had initially experienced higher excess mortality tended to exhibit less pronounced increases. Overall, a consistent pattern of excess mortality across federal states emerged during the first two years of the pandemic, suggesting that the factors driving excess mortality in the first two pandemic years remained stable both in their effects across time and in their regional effects over time.

In the third year of the pandemic, excess mortality patterns shifted markedly, characterized by three key changes: (i) a sharp increase in excess mortality was observed across nearly all federal states; (ii) this general rise was accompanied by a reduction in inter-state variability; and (iii) the distribution pattern of excess mortality changed substantially, with different federal states exhibiting the highest levels of excess mortality compared to the first two years. This pattern suggests that a new factor contributing to excess mortality emerged in the third year, affecting all federal states equally, but with a different distribution pattern across federal states compared to the factors driving excess mortality in the first two pandemic years.

Given the results regarding the regional distribution pattern and the trajectory of excess mortality, two fundamental questions arise: The first question concerns which factors may have been responsible for excess mortality in the first two years of the pandemic, while the second question addresses which additional factors may have emerged in the third pandemic year and contributed to the sharp increase and the altered regional distribution of excess mortality.

Both of these questions are addressed in the following sections. Before proceeding, however, we would like to clarify one point: As our expertise lies in the methodologically sound estimation of excess mortality and the statistically robust analysis of its relationship with the explored state-specific indicators, the aim of the following section is to provide a well-founded overview of the underlying data and patterns. These insights can then serve as a basis for experts in the fields of medicine and public health to investigate the causes that may have led to the observed patterns.

### 5.1. Excess mortality during the first two pandemic years

Regarding possible explanations for the variation in excess mortality during the first two pandemic years, the correlational results appear to provide a clear answer: Since excess mortality in a federal state was strongly positively correlated with the reported numbers of COVID-19 deaths and infections, the observed variation in excess mortality across federal states during the first two pandemic years appears to be mainly attributed to the varying extent of COVID-19 impact in different federal states. However, a closer look reveals some inconsistencies.

Although the number of reported COVID-19 deaths is highly correlated with the number of observed excess mortality deaths, it is important to note that the number of reported COVID-19 deaths substantially exceeds the number of observed excess mortality deaths: In the first pandemic year, reported COVID-19 deaths were 3.5 times higher than the observed excess mortality deaths, and in the second pandemic year, twice as many COVID-19 deaths were reported compared to the observed excess mortality deaths. Notably, in both years, at the same time, fewer non-COVID-19-related deaths were reported than statistically expected.

This surprising pattern may have occurred owing to two possible reasons. First, it could be that the measures taken against COVID-19 also reduced the number of non-COVID-19-related deaths, and that the reported COVID-19 deaths are all true excess deaths. However, this possibility is unlikely given the observed correlations between the decline in non-COVID-19-related deaths observed in a federal state and the strength of the measures taken. In the first pandemic year, a zero correlation is observed (r=0.06, p=0.823), and in the second pandemic year, even a tendentially negative correlation is observed (r=−0.38, p=0.152), that is, the stronger the measures taken, the smaller the decline in the number of observed non-COVID-19-related deaths. It is therefore more likely that a second possibility is the case: COVID-19 may have replaced other commonly occurring causes of death. One possible mechanism may be that the spread of to the SARS-CoV-2 virus inhibited the viral reproduction of other common viruses (e.g. Deleveaux *et al.* [26]), another possible mechanism may be that a certain proportion of deaths reported as ‘COVID-19 death’ may in fact have resulted from other causes (e.g. von Stillfried [27]).

Regardless of which of the two possibilities primarily accounts for the observed pattern, it is important to note that the excess mortality observed in Germany during the first two pandemic years was on par with the excess mortality seen in previous severe influenza seasons, such as the 2017/2018 flu season, during which an estimated 25 100 deaths occurred owing to influenza (RKI [28]). This suggests that the reported number of COVID-19 deaths significantly overestimated the true burden of COVID-19 on excess mortality, which remained within the range typically seen during major influenza waves in Germany.

Another notable observation emerges when comparing the trajectory of excess mortality deaths with the trajectory of reported COVID-19 deaths over the first two years of the pandemic. While excess mortality slightly increased from the first to the second pandemic year, the number of reported COVID-19 deaths strongly decreased. This inverse trend suggests that, despite the strong positive correlation between the two variables, COVID-19 alone cannot fully explain the excess mortality observed in the second year of the pandemic. That is, the increase in excess mortality during that period must be attributed, at least in part, to the emergence of additional factors beyond COVID-19.

Among the state-specific indicators examined, the only newly introduced factor in the second year of the pandemic is the COVID-19 vaccination. Indeed, a strong negative correlation is observed between excess mortality in the second year of the pandemic and the vaccination rate across federal states. At first glance, given the observation that excess mortality was lower in states with higher vaccination rates, it seems that COVID-19 vaccinations contributed to a reduction in excess mortality. However, the fact that vaccination rates were also negatively correlated with excess mortality in the first year of the pandemic to exactly the same extent—when vaccinations could not have had a meaningful impact—argues strongly against such an interpretation. Instead, this correlation suggests that in federal states where excess mortality was less pronounced in the first year, vaccination rates were particularly high in the following second year. Since excess mortality remained relatively stable during the first two pandemic years, it is highly likely that the negative association between vaccination rates and excess mortality reflects the influence of a time-invariant third variable. This variable probably reduced excess mortality in certain federal states while simultaneously increasing vaccination rates in those same states.

Such an interpretation is supported by a change score analysis that controls for time-invariant, unobserved third variables, such as demographic structure, healthcare capacity or baseline health status. The analysis revealed that the increase in excess mortality from the first to the second pandemic year was higher in federal states with higher vaccination rates, suggesting that the vaccinations may have increased rather than decreased excess mortality. From a purely statistical perspective, this could indeed suggest that the newly emerging factor contributing to excess mortality in the second year of the pandemic is the COVID-19 vaccination. However, when adjusting for excess mortality in the first pandemic year, this relationship reversed, suggesting that higher vaccination rates were linked to smaller increases in excess mortality. However, given the high negative correlation between excess mortality in the first pandemic year and vaccination rates, this analysis should be interpreted cautiously, as shared variance makes it challenging to attribute the effect to either factor independently.

### 5.2. Excess mortality during the third pandemic year

Although COVID-19 is a plausible explanation for excess mortality in the first and, to some extent, the second pandemic year, this does not account for the strong increase in excess mortality observed in the third pandemic year. Notably, instead of increasing, the number of reported COVID-19 deaths decreased from the second to the third pandemic year. In addition, no significant correlation is observed between the number of reported COVID-19 deaths and excess mortality in the third pandemic year. At first glance, one might assume that the still significant positive correlation between reported SARS-CoV-2 infections and excess mortality in the third year of the pandemic could suggest that COVID-19 might be a potential explanation for the significant increase. However, this possibility is ruled out by the fact that the number of SARS-CoV-2 infections also decreased rather than increased from the second to the third year.

The data also appear to rule out long-term effects of SARS-CoV-2 infections, such as Long COVID, as a contributing factor to excess mortality during the third pandemic year. If Long COVID were a significant contributing factor, one would expect positive correlations between excess mortality and the number of SARS-CoV−2 infections in the first and second years of the pandemic. However, our data reveal the opposite: higher infection rates in the first (r=−0.42, p=0.103) and second (r=−0.42, p=0.102) years are associated with lower excess mortality in the third year. These inverse relationships suggest that Long COVID is not a primary driver of excess mortality in the third pandemic year.

Regarding the examined state-specific indicators, the only relevant correlational pattern with excess mortality in the third year of the pandemic is observed in relation to the COVID-19 vaccination rates. Surprisingly, all correlations suggest a positive rather than negative association between vaccination rates and excess mortality in the third pandemic year. Specifically, excess mortality in the third year of the pandemic was highest in the federal states with the highest vaccination rates. This relationship remains significant when change scores are calculated to control for time-invariant third variables, indicating that excess mortality from the second to the third year increased most in the states with the highest vaccination rates. This relationship persists even when controlling for the extent of excess mortality in a state during the first two years of the pandemic.

A similar pattern is observed for the relationship between the vaccination rate of a federal state and COVID-19-related deaths in the third year of the pandemic. Specifically, from the second to the third year of the pandemic, not only did the number of reported COVID-19 deaths in a state decrease less sharply the higher the vaccination rate, but also the SARS-CoV-2 case fatality rate decreased less sharply the higher the vaccination rate. Notably, when dividing the federal states into two groups based on higher versus lower vaccination rates, those with higher vaccination rates showed, on average, an increase rather than a decrease both in the number of reported COVID-19 deaths and in the case fatality rate in the third pandemic year.

Taken together, the vaccination pattern closely mirrors the pattern observed in the increase in excess mortality. As outlined earlier, the excess mortality trend is characterized by three key features: (i) a sharp increase in excess mortality starting from the second year of the pandemic, (ii) a significant reduction in the variance across federal states, and (iii) a shift in the ranking order of the federal states with the highest excess mortality. These same three characteristics are also reflected in the vaccination pattern: (i) from the second year of the pandemic onward, COVID-19 vaccinations became a new factor in all federal states, (ii) a large proportion of the population was vaccinated in each federal state, that is, all states were strongly affected by this factor, and (iii) the federal states with the highest vaccination rates have risen in the ranking order of the federal states with the highest excess mortality.

### 5.3. Comparison with other studies

Interestingly, a similar pattern of correlations between vaccination rates and excess mortality has also been observed in comparable studies conducted on excess mortality in Germany and in other countries.

As mentioned in the introduction, a recent study estimated excess mortality in 29 European countries from 2020 to 2023 and examined associations with various country-specific indicators, finding that excess mortality in the period 2020−2023 was positively correlated with the percentage of the populations living in poverty and the Gini index, and negatively correlated with the GDPs per capita, health expenditures and COVID-19 vaccination rates (Pizzato *et al.* [10]). The authors provided estimates of annual excess mortality in a table. And indeed, when examining these yearly data, a pattern emerges that closely mirrors the one observed in the present study. Vaccination rate was also already negatively correlated with excess mortality in 2020, when vaccines were not yet administered (r=−0.36), and the correlation between vaccination rate and excess mortality changed from negative to positive over the course of the pandemic (2021: r=−0.81; 2022: r=−0.28; 2023: r=0.28). Calculating change scores showed that vaccination rate was negatively correlated with the change in excess mortality from 2020 to 2021 (r=−0.70) but positively correlated with the change in excess mortality from 2021 to 2022 (r=0.81) and from 2022 to 2023 (r=0.60).

A similar pattern was also observed in a study on excess mortality in Austria (Reitzner [29]), where vaccination rate was also already negatively correlated with excess mortality in the first year of the pandemic (r=−0.57), and the correlation between vaccination rate and excess mortality decreased over the course of the pandemic (second pandemic year: r=−0.60; third pandemic year: r=−0.26). Calculating change scores showed that the increase in excess mortality from the first to the third pandemic year was positively correlated with a federal state’s vaccination rate (r=0.61).

In a small-scale study [30] of mortality in Frankfurt am Main, Germany, from 2020 to 2023, findings consistent with the present analysis indicated that the high excess mortality observed in the third pandemic year could not be attributed to COVID-19. Instead, most of the excess mortality during that year was associated with a severe influenza wave in the final 6 weeks, which produced higher excess mortality in Frankfurt than any wave of the SARS-CoV-2 pandemic in the city. This raises the question of whether such a pattern can be generalized to Germany as a whole. If this were the case, one would expect a positive correlation between state-level excess mortality in the third pandemic year and the number of influenza infections during the same period. However, data from the RKI [31], which reports influenza infections separately by federal state, show the opposite: a negative correlation between influenza infection rate and excess mortality in the third pandemic year (r=−0.44, p=0.11).

### 5.4. Further correlation patterns

Beyond the observed associations between excess mortality, COVID-19-related factors, and vaccination rates, only one other state-level indicator examined revealed a notable correlation pattern: trust in institutions. Trust in institutions showed significant correlations with both vaccination behaviour and mortality trends. Specifically, vaccination rates were higher in federal states with greater levels of institutional trust, consistent with prior findings identifying institutional trust as a key determinant of vaccination behaviour (e.g. Bajos *et al.* [32]). Furthermore, replicating findings by Zaki *et al.* [33], institutional trust was negatively associated with excess mortality in the first year of the pandemic. However, this pattern reversed in subsequent years: trust in institutions was positively correlated with the increase in excess mortality from the first to the second year (r=0.55, p=0.029) and from the second to the third year (r=0.59, p=0.017). In other words, the higher the level of institutional trust, the greater the increase in excess mortality over time.

A simple mediation analysis was performed to investigate whether trust in institutions predicted the increase in excess mortality from the second to the third pandemic year and whether the effect was mediated by vaccination rate. The analysis revealed that trust in institutions significantly predicted excess mortality (β=0.46, t(13)=3.04, p=0.009). When vaccination rate was added as a mediator, trust in institutions significantly predicted vaccination rate (β=1.02, t(13)=3.85, p=0.002), which in turn significantly predicted the increase in excess mortality (β=0.61, t(13)=7.90, p<0.001). However, after accounting for the mediation by vaccination rate, trust in institutions no longer significantly predicted excess mortality (β=−0.16, t(13)=−1.95, p=0.073), indicating a full mediation of the relationship between trust in institutions and excess mortality by vaccination rate.

With regard to the other state-specific indicators examined, no systematic correlation patterns were observed. Only a few small, time-limited correlations emerged. In the second pandemic year, excess mortality showed a moderate correlation with both mean age and the proportion of people in need of care—two variables that are themselves highly correlated. In the third pandemic year, excess mortality was moderately correlated with the poverty rate. Given the high number of correlations tested, along with their moderate strength and sporadic occurrence, it is likely that these correlations are due to chance rather than reflecting true effects.

Notably, no significant association was found between the stringency of pandemic measures and excess mortality, reported COVID-19 deaths, or SARS-CoV-2 infections in any of the three pandemic years. This absence of measurable effects contrasts with expectations and aligns with findings from a large-scale multiverse analysis of 16 types of government responses across 181 countries (Bendavid & Patel [34]). Among nearly 100 000 analytic models, only 42% suggested improved outcomes with stricter measures, while 58% indicated worse outcomes. The study highlights the analytical flexibility inherent in such evaluations and concludes that strong claims about the effectiveness of government interventions lack consistent empirical support.

## 6. Conclusion

Based on state-of-the-art actuarial methods, the present study demonstrates that Germany experienced moderate average excess mortality during the first two years of the pandemic, with substantial and temporally stable regional variation across federal states. In the third pandemic year, excess mortality rose sharply, regional variation diminished and the pattern of the most affected federal states shifted markedly.

The strong correlation between excess mortality and reported COVID-19 deaths and infections during the first two pandemic years suggests that regional differences in COVID-19 burden may account for much of the observed variation. However, the increase in excess mortality during the second year, despite a decrease in reported COVID-19 deaths, indicates that COVID-19 alone cannot fully explain excess mortality in that period. This discrepancy suggests the emergence of an additional factor contributing to excess mortality in the second pandemic year.

The sharp rise in excess mortality in the third pandemic year is unlikely to be attributable to COVID-19, given the continued decline in reported COVID-19 deaths and the absence of a correlation with the regional excess mortality variation. Instead, a surprisingly strong positive correlation emerges with the vaccination rate of a federal state, a correlation that persists even when controlling for prior levels of excess mortality. Moreover, the higher the vaccination rate, the smaller the decline in reported COVID-19 deaths and the SARS-CoV-2 case fatality rate from the second to the third pandemic year. In states with higher vaccination rates, there is even a slight increase in both indicators rather than a decrease.

From the perspective of the medical studies on vaccine effectiveness, the observed correlation pattern is highly surprising. For instance, according to a large meta-analysis covering the period up to the end of December 2022, across all SARS-CoV-2 strains, vaccine effectiveness directly after vaccination seems to be 91% for mortality, with a slight decline to 86% in the long run (Wu *et al.* [35]). If vaccine effectiveness were truly as high as assumed, it would be difficult to explain why the federal states with the highest vaccination rates also show the strongest increase in excess mortality, along with an increase—rather than a decrease as observed in the federal states with lower vaccination rates—in the number of reported COVID-19 deaths and the SARS-CoV-2 case fatality rate. This is particularly striking given that in the states with the highest vaccination rates, more than 97% of the population aged 60 and older were at least fully vaccinated.

When interpreting studies on vaccine effectiveness, it is important to note that most were observational and lacked random assignment to vaccinated and unvaccinated groups. Such designs are more prone to bias and generally yield less reliable estimates of causal effects (e.g. Jefferson [36]). Against this background, it is notable that in the large randomized controlled trials of COVID-19 vaccines, no beneficial effect on all-cause mortality was observed. For example, in the 6 month interim report of adverse events from the Pfizer-BioNTech trial, 21 deaths were reported in the vaccine group (including two unblinded participants from the original placebo group who were later vaccinated) compared with 17 deaths in the placebo group (Michels *et al.* [37]). Consistent with the correlational patterns observed in the present study, this finding suggests not a mortality benefit but rather a possible excess of deaths among vaccinated participants. A similar pattern was observed for serious adverse events: in a reanalysis of the original trial data, statistically significant increases in serious adverse events occurred in the vaccine group, with the excess risk more than four times greater than the reduction in risk of COVID-19 hospitalization in the placebo group (Fraiman *et al.* [38]). Taken together, the all-cause mortality findings from randomized trials and the correlational patterns observed in the present study underscore the need for rigorous investigation into possible unintended consequences of vaccination.

However, it is important to note that although the correlation analysis observed in the present study yields a clear pattern, and although the applied analytical approach accounts for time-invariant third variables and prior levels of excess mortality, the possibility remains that a hidden confounding factor—coincidentally correlated with vaccination rates—may underlie the observed increase in excess mortality. Furthermore, it is important to emphasize that the reported associations are correlational in nature and do not imply that differences in vaccination rates between federal states causally account for the observed differences in excess mortality. In this context, it is particularly important to reiterate, as noted at the beginning of the discussion, that the conclusions drawn here are based solely on a statistical perspective and not on the medical literature regarding the efficacy of COVID-19 vaccines. Accordingly, the reported correlational patterns should be regarded as a starting point for experts in medicine and public health to further explore potential underlying mechanisms.

## Data Availability

Only data sets from official sources were used; the respective sources are provided in the References. Further inquiries can be directed to the corresponding author. Supplementary material is available online [39].
